# Prevalence of abnormal umbilical arterial flow on Doppler ultrasound in low-risk and unselected pregnant women: a systematic review

**DOI:** 10.1186/s12978-021-01088-w

**Published:** 2021-02-12

**Authors:** Joshua P. Vogel, Valerie Vannevel, Gianna Robbers, George Gwako, Tina Lavin, Abiodun Adanikin, Tsakane Hlongwane, Robert C. Pattinson, Zahida P. Qureshi, Olufemi T. Oladapo

**Affiliations:** 1 Maternal, Child and Adolescent Health Program, Burnet Institute, 85 Commercial Road, Melbourne, 3000 Australia; 2 South African Medical Research Council/University of Pretoria Maternal and Infant Health Care Strategies Unit, Department of Obstetrics and Gynaecology, University of Pretoria, Unit Private Bag X323 Arcadia, Pretoria, 0007 South Africa; 3 Department of Obstetrics and Gynaecology, University of Nairobi, Nairobi, Kenya; 4 School of Population and Global Health, University of Western Australia, Hackett Drive, Crawley, Perth, Australia; 5 UNDP/UNFPA/UNICEF/WHO/World Bank Special Programme of Research, Development and Research Training in Human Reproduction (HRP), Department of Sexual and Reproductive Health and Research, World Health Organization, Geneva, Switzerland

**Keywords:** Doppler ultrasound, Antenatal care, Pregnancy, Stillbirth

## Abstract

**Background:**

While Doppler ultrasound screening is beneficial for women with high-risk pregnancies, there is insufficient evidence on its benefits and harms in low- and unselected-risk pregnancies. This may be related to fewer events of abnormal Doppler flow, however the prevalence of absent or reversed end diastolic flow (AEDF or REDF) in such women is unknown. In this systematic review, we aimed to synthesise available data on the prevalence of AEDF or REDF.

**Methods:**

We searched PubMed, Embase, CINAHL, CENTRAL and Global Index Medicus with no date, setting or language restrictions. All randomized or non-randomized studies reporting AEDF or REDF prevalence based on Doppler assessment of umbilical arterial flow > 20 weeks’ gestation were eligible. Two authors assessed eligibility and extracted data on primary (AEDF and REDF) and secondary (fetal, perinatal, and neonatal mortality, caesarean section) outcomes, with results presented descriptively.

**Results:**

A total of 42 studies (18,282 women) were included. Thirty-six studies reported zero AEDF or REDF cases. However, 55 AEDF or REDF cases were identified from just six studies (prevalence 0.08% to 2.13%). Four of these studies were in unselected-risk women and five were conducted in high-income countries. There was limited evidence from low- and middle-income countries.

**Conclusions:**

Evidence from largely observational studies in higher-income countries suggests that AEDF and REDF are rare among low- and unselected-risk pregnant women. There are insufficient data from lower-income countries and further research is required.

## Plain language summary

Doppler ultrasound can be used during pregnancy to determine how well blood is flowing through the umbilical cord. When this blood flow is restricted, absent or even reversed, the health of the baby can be threatened. Poor umbilical blood flow can lead to a baby experiencing growth restriction. If the flow is absent or reversed, the baby may die. In this review, we aimed to determine how often pregnant women experience abnormal umbilical flow during pregnancy, in particular the occurrence of absent or reversed flow. We were interested in how often this occurred in women who had a singleton, low-risk pregnancy (i.e. women without significant medical, obstetric or fetal complications of pregnancy). We found 42 studies reporting on over 18,000 women, mostly from high-income countries. Across all studies, 55 women experienced absent of reversed blood flow in the umbilical artery, all of which occurred in just six studies. However we found limited evidence from low- and middle-income countries, where rates of growth restriction and preventable stillbirth are quite high. Further research on abnormal umbilical blood flow in pregnant women in low- and middle-income countries is required.

## Introduction

An estimated 2.6 million stillbirths occur every year worldwide, 98% of which occur in low- and middle-income countries (LMICs) [1, 2]. Intrauterine growth restriction (IUGR) describes a pathological inhibition of fetal growth that prevents the fetus from attaining its growth potential [3]. The incidence of IUGR is difficult to estimate, varying between populations, settings and definitions used [4]. However, the prevalence of small-for-gestational-age (many of whom are growth restricted) is nearly 20% in low- and middle-income countries [5]. It is a well-established risk factor for stillbirth, and can increase the risk of stillbirth by up to eightfold [6, 7]. IUGR is also associated with higher rates of neonatal death, perinatal morbidity and non-communicable diseases (such as diabetes) into adulthood [8–13]. Placental insufficiency is the leading cause of IUGR, mostly due to poor uteroplacental blood flow, placental thrombi and infarctions [14, 15]. Despite the high prevalence of IUGR in many LMICs (up to 19% of live births in LMICs are small-for-gestational age), it is often not detected during routine antenatal care [5, 7, 16, 17]. Undetected IUGR increases the risk of serious fetal complications, making the detection and management of IUGR prior to birth crucial to preventing adverse perinatal outcomes [18].

Doppler ultrasound can be used during pregnancy to detect blood flow within the blood vessels of the umbilical cord as a proxy measure of placental function [19]. When blood flow is absent (i.e. absent end diastolic flow, AEDF) or reversed (i.e. reverse end diastolic flow, REDF), urgent referral for specialist care is needed to avert a perinatal death [20]. The use of Doppler ultrasound to assess umbilical blood flow in women with high-risk pregnancies has been shown to prevent perinatal deaths; its use in these trials ranged from 24 to 40 weeks gestation [19]. Conversely, a Cochrane review on the use of Doppler ultrasound in low-risk or unselected populations of pregnant women identified only five trials (involving 14,185 women) from high-income country settings [21]. The authors concluded that there was insufficient evidence on whether the use of routine umbilical artery Doppler ultrasound in low-risk or unselected populations benefits either the mother or baby [21]. WHO does not currently recommend routine Doppler ultrasound for women with no identifiable risk factors in pregnancy, however the WHO guideline panel has stated that the value of routine application of single Doppler ultrasound assessment of the fetal blood vessels during the third trimester needs more rigorous evaluation, particularly in LMICs where stillbirth rates are high and Doppler ultrasound is not routinely unavailable [22].

To further evaluate the value of Doppler ultrasound assessment of umbilical flow in pregnancy particularly in LMICs where stillbirth rates are unacceptably high, an estimate of the likely prevalence of abnormal Doppler flow (especially AEDF and REDF) is required. However, this estimate is currently unknown. This systematic review aims to fill this knowledge gap by summarising all available data on the prevalence of abnormal umbilical artery flow indices (AEDF or REDF) in populations of low-risk or unselected pregnant women.

## Methods

The review protocol was registered on PROSPERO (CRD42020161980) and reported according to the Preferred Reporting Items for Systematic Reviews and Meta-Analyses (PRISMA) checklist (see Additional file 1: Appendix S1) [23]. As a systematic review of published studies, ethical approval was not required.

### Eligibility criteria

Eligible studies were primary research studies that reported the prevalence of AEDF and/or REDF amongst low-risk or unselected-risk pregnant women with singleton pregnancies. Randomized or non-randomized study designs (including cohort or cross-sectional studies, and randomized or non-randomized interventional studies) were eligible for inclusion, though case reports, case series and commentaries were not eligible. Conference abstracts were eligible provided that sufficient information was available for data extraction and quality assessment. The primary outcomes of interest were the prevalence of AEDF or REDF. Secondary outcomes included mortality (stillbirth, perinatal death, early neonatal death, neonatal death) and mode of birth (percentage of caesarean births).

Studies were eligible if they included either “unselected-risk” pregnant women (defined as women attending non-specialist antenatal care clinics, but have not been classified as being low- or high-risk based on local clinical guidelines, or as defined by study authors) or low-risk women (defined as women attending non-specialist antenatal care clinics, who have been classified as “low-risk” according to local clinical guidelines, or defined as low-risk by the study authors). Studies involving only high-risk women (regardless of how they were defined) were not eligible. If studies included mixed populations, they were included only if data could be extracted separately for low- and/or unselected-risk populations. Studies were eligible if umbilical arterial flow was assessed by Doppler ultrasound, regardless of whether this was continuous or pulsed-wave Doppler. We excluded studies where Doppler assessment was performed only in the first 20 weeks of pregnancy—this threshold was used as it reflects a when a consistently positive umbilical arterial flow can be expected. We also excluded studies of multiple pregnancies, post-term pregnancies, or where Doppler assessment was done during labour or immediately prior to caesarean section or labour induction.

### Literature searching, data collection and analysis

We searched PubMed, Embase, CINAHL, Cochrane CENTRAL and Global Index Medicus for relevant studies with no date, setting or language restrictions (initial search 19 December 2017, updated search 10 January 2020, see Additional file 2: Appendix S2 for search strategy). At least two review authors independently screened all titles and abstracts, assessed full texts of potentially eligible studies and extracted data (disagreements were resolved by discussion). Covidence software was used for title, abstract and full text screening [24].

Extracted data from each study included characteristics of the study (design, year, country, sample size, study eligibility criteria), participants (including maternal risk profile) and Doppler assessment. Where necessary, we contacted authors of the included studies for additional information on the primary outcomes. Characteristics of studies and populations and prevalence of review outcomes were reported descriptively (SPSS version 26) [25]. We planned but could not perform a meta-analysis due to significant methodological and reporting heterogeneity and sparseness of primary outcome events. Countries were classified into low, low-middle, upper-middle and high-income countries based on the current World Bank classification [26].

As this review include different study designs, we used different tools to assess methodological quality. Observational studies were assessed using the relevant iteration of the Newcastle–Ottawa scale for case–control, cohort or cross-sectional studies [27, 28]. Randomised controlled trials were assessed using the Cochrane risk of bias tool.

## Results

**Fig. 1:**
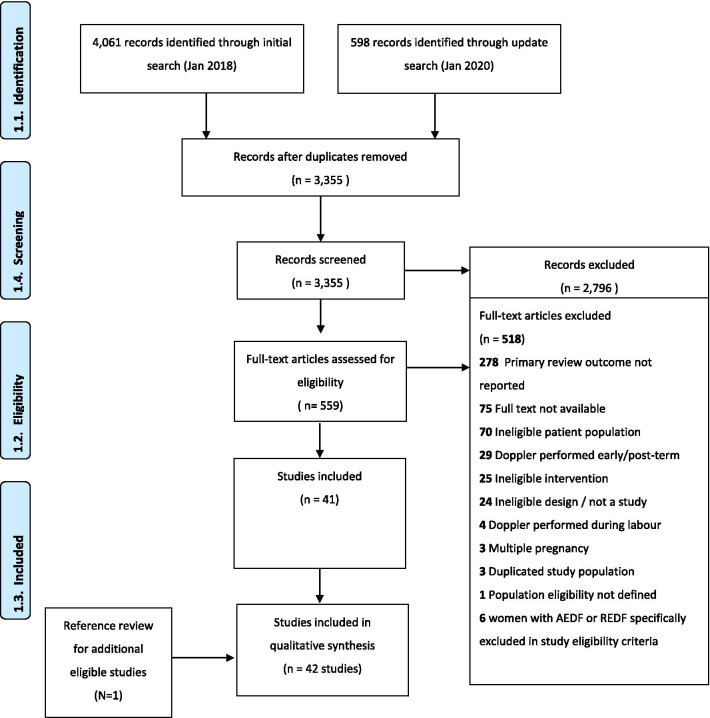
PRISMA flowchart

A total of 3355 unique citations were screened, of which 2796 were excluded (Fig. 1). Of the 559 citations included for full text review, 518 were excluded. A total of 42 studies were included, from which data on 18,282 women were extracted (Additional file 3: Appendix S3). Included studies were mostly observational designs (38 studies), though four randomized controlled trials were included (Table 1). A total of 37 studies were conducted in high income or upper-middle income countries, four studies were conducted in low-middle income countries and one was a multi-country study was conducted in Brazil, Kenya and the UK (zero studies in low-income countries) [29]. The year of publication ranged from 1983 to 2020. Twenty studies did not specify the years in which data were collected, though for studies that did specify, data collection occurred between 1987 and 2017.

**Table 1:** Characteristics of included studies

Study design	N	%
Case–control study	7	16.7
Cross-sectional study	12	26.2
Cohort study	19	45.2
Randomized controlled trial	4	9.5
Country income level		
High-income countries	24	57.1
Upper-middle income countries	13	31.0
Low-middle income countries	4	9.5
Low-income countries	0	0.0
Multiple^a^	1	2.1

^a^One study was a multi-country study conducted in Brazil (upper-middle income country), Kenya (low-middle income country) and the UK (high-income country)

In total, 36 studies included low-risk pregnant women only, while six studies included unselected-risk women (though these definitions varied across studies) (Table 2). Where it was reported, studies had excluded women with anomalous pregnancies. Sample sizes from which data were extracted ranged from 14 to 2868 women; 24 studies had a sample of less than 200 women. The mean maternal age (reported in 27 studies) ranged from 19.9 to 32 years. The mean gestational age of the study population was only specified in 22 studies, ranging from 20 to 40 weeks (in 8 studies, the mean gestational age was 38 to 40 weeks). Eight studies did not specify the type of Doppler used, though 6 used continuous-wave, 6 used pulsed-wave and 1 study used continuous or pulsed wave (practice varied between participating centres [30]). Overall, 6 of 7 case–control studies, 9 of 12 cross-sectional studies and all cohort studies were assessed as satisfactory, good or very good quality (Additional file 4: Appendix S4). The four trials were assessed as low or unclear risk of bias across all domains, except for lack of blinding of participants, personnel and outcome assessment in three of four trials [30–32].

**Table 2:** Characteristics of women and Doppler assessments in included studies

Study population	N (studies)	%
Maternal risk		
Low-risk women only	36	85.7
Unselected women	6	14.3
Doppler assessments		
Doppler ultrasound of umbilical artery only reported	15	35.7
Doppler ultrasound of umbilical artery plus other Doppler investigations reported^a^	27	64.2
Type of Doppler		
Colour Doppler	12	28.6
Continuous-wave Doppler	6	14.3
Pulsed-wave Doppler	6	14.3
Pulsed-wave Doppler; colour Doppler	9	21.4
Pulsed-wave and continuous-wave Doppler	1	2.1
Doppler (not otherwise specified)	8	19.0

^a^Fetal cerebral circulation, fetal aorta, fetal renal arteries, umbilical vein, ventricular outlets, femoral vessels, uterine/placental vessels

Across all 42 studies (18,282 women), 36 studies had zero AEDF events, while six studies reported a total of 55 cases of AEDF or REDF. Forty-eight of these cases were AEDF only and seven were AEDF or REDF, reported in a 1993 study in South Korea by Yoon et al. [33]. These six studies reported a prevalence ranging from 0.08 to 2.13% (Table 3) [31–37]. They were generally larger samples (ranging from 328 to 2868 women)—four studies were in unselected-risk women and five were conducted in high-income countries. The seventh study by Nkosi et al. was conducted in South Africa (an upper-middle income country) and contributed 38 of the AEDF cases we identified [37]. They used a low-cost, handheld, continuous flow Doppler apparatus as a routine screening tool for assessing umbilical vessel flow in 2868 unselected-risk women attending two community health centres for antenatal care. The highest prevalence was reported by Yoon et al., who reported a prevalence of AEDF or REDF of 2.13% amongst 328 unselected-risk women in South Korea [38]. The four studies (898 women) conducted in lower middle-income countries (Bangladesh, Papua New Guinea, Nigeria and Tunisia) did not report any cases of AEDF or REDF [39–42]. Similarly, a three-country prospective cohort study of 431 healthy, low-risk women by Drukker et al. in Brazil, Kenya and the UK identified no AEDF cases in any country [29].

**Table 3:** Studies with a prevalence of absent or reversed end diastolic flow greater than zero

Study	Design	Study population	AEDF prevalence
Low risk women			
Souka 2012 [36]	Cross-sectional study	2189 low-risk women in Greece	1/1289 (0.05%)
Mason 1993 [32]	RCT	863 low-risk, nulliparous women in the UK (Doppler arm only)	2/863 (0.23%)
Unselected risk women			
Davies 1992 [31]	RCT	1246 unselected-risk women in the UK (Doppler arm only)	1/1246 (0.08%)
Beattie 1989 [34]	Cohort	2097 unselected-risk women in USA	6/2097 (0.29%)
Nkosi 2019 [37]	Cohort	2868 unselected-risk women in South Africa	38/2868 (1.32%)
Yoon 1993 [38]	Cohort	328 unselected women in South Korea	7/328 (2.13%)^a^

Results of all included studies are in Additional file 3: Appendix S3

^a^Defined as AEDF or REDF

The CS rate for the population of interest was generally not reported (25 studies). Where it was reported, it ranged from 2.9 to 57.1% (Additional file 3: Appendix S3). Similarly, in most studies, stillbirth (30 studies), early neonatal death (36 studies), perinatal death (31 studies) and neonatal death (34 studies) were not reported for the population of interest. Where these rates were reported, they were very low, except for the study by Yoon et al. that reported seven cases of AEDF or REDF, 10 stillbirths and 18 perinatal deaths among 328 unselected-risk women [38].

## Discussion

This systematic review identified the prevalence of severe umbilical arterial flow abnormalities (AEDF or REDF) amongst studies involving low-risk or unselected pregnant women to be 0% to 2.13%. The available evidence is largely from observational studies of reasonable quality conducted in high or upper-middle income countries. The 55 cases of AEDF or REDF were identified in six studies, of which four studies were in unselected-risk women.

End-diastolic flow is one of several parameters that can be measured to assess fetal haemodynamics during pregnancy. The identification of absent or reversed flow in the umbilical artery during the second half of pregnancy is an indication for urgent referral, steroid administration and typically immediate delivery due to its association with high perinatal morbidity and mortality [43]. While Doppler ultrasound assessment in women with high-risk pregnancies has been shown to prevent perinatal deaths [19], its effectiveness as a routine screening tool is dependent on the underlying prevalence of abnormal blood flow in these high-risk women, which is considerably higher than in the general obstetric population. For example, the largest trial in the Cochrane review of Doppler ultrasound in high-risk women was conducted by Johnstone and colleagues in the UK [19, 44]. They recruited 2289 women who attended the hospital during the study period, of whom 8% had an RI above the normal range (i.e. more than 2 standard deviations beyond the mean) on umbilical artery Doppler assessment. Our findings suggest that in low-risk obstetric populations in high-income countries, the prevalence of AEDF or REDF ranges from zero to very low, restricting its use as a routine screening tool. This could potentially explain why the Cochrane review on use of Doppler ultrasound in women with normal pregnancies did not find clinical benefit for this intervention in five trials (14,185 women) in high-income country settings [21].

Our review identified only four studies (898 women) conducted exclusively in low-middle income countries, which is an insufficient sample size to confirm the prevalence of AEDF or REDF in a low- or unselected-risk population of pregnant women. However, the study conducted by Nkosi et al. in South Africa with 2868 unselected-risk women identified an AEDF prevalence of 1.29%, which is suggestive that the prevalence of AEDF in some lower-resource settings may be higher than is appreciated. Furthermore, in this study women with raised resistance index on umbilical arterial flow assessment were referred to higher-level obstetric care services, resulting in a 42% risk reduction in perinatal mortality [37]. This suggests there may yet be a role for Doppler ultrasound in preventing stillbirths amongst women with low-risk pregnancies in LMIC settings. However, further research (including randomised trials) will be required to evaluate the benefits and possible harms of using Doppler flow assessment in low-risk antenatal care in LMICs. In addition, further synthesis and primary studies on the prevalence of other, earlier indicators of abnormal flow (such as raised resistance and pulsatility indices) which precede AEDF. Such research can yield important insights into how women at risk of IUGR may be identified.

### Strengths and limitations

Strengths of this review included a broad search strategy across multiple databases with minimal restrictions. However, the findings of this review are reliant on the quality of the underlying studies, some of which were conducted as uncontrolled or retrospective analyses of routinely collected observational data. One limitation is the variation in definitions of maternal baseline risk between different studies and settings—in several instances, the eligibility criteria were not well-described. Other limitations include poor or inconsistent reporting of study parameters such as precise details of method of Doppler assessment.

## Conclusion

This review has shown that the prevalence of AEDF or REDF in populations of low- or unselected-risk pregnant women is zero or very low. However, there is limited evidence available on AEDF or REDF prevalence in these populations of women in LMICs, where the burden of fetal growth restriction and stillbirth is unacceptably high. Further research is required to determine the prevalence of AEDF or REDF in such settings, which could be used to inform further research on the effectiveness of integrating Doppler assessments into routine antenatal care in LMICs. To address this knowledge gap, WHO is sponsoring an ongoing multi-country study on the prevalence of abnormal Doppler flow.

## Supplementary Information

**Additional file 1: Appendix S1.** PRISMA checklist.

**Additional file 2: Appendix S2.** Search strategy.

**Additional file 3: Appendix S3.** Included studies.

**Additional file 4: Appendix S4.** Quality assessments.

## Data Availability

The dataset is provided in additional files.

## Abbreviations

AEDF: Absent end diastolic flow
IUGR: Intrauterine growth restriction
LMICs: Low- and middle-income countries
REDF: Reversed end diastolic flow
WHO: World Health Organization
