# Supplementary material for: Prevalence of abnormal umbilical arterial flow on Doppler ultrasound in low-risk and unselected pregnant women: a systematic review
Source: Reprod Health. 2021 Feb 12;18:38. doi: 10.1186/s12978-021-01088-w (PMC7881445; doi:10.1186/s12978-021-01088-w)
Supplement: Supplementary file 2 — Additional file 2: Appendix S2. Search strategy. [file 12978_2021_1088_MOESM2_ESM.docx]

**Appendix 2. Search strategy**

**PubMed (19 December 2017, updated 10 January 2020)**

| **Concept** | **Search string** | **Results (19.12.17)** | **Results (10.01.20)** |
| --- | --- | --- | --- |
| 1-umbilical | "Umbilical Arteries"[Mesh] OR "Umbilical Cord"[Mesh] OR "Umbilical Veins"[Mesh] OR "Single Umbilical Artery"[Mesh] OR“umbilical vessels”[Tiab] OR “umbilical cord” [Tiab] OR “umbilical cable” [Tiab] OR “umbilical artery” [Tiab] OR “umbilical arteries” [Tiab] OR “umbilical circulation” [Tiab] OR “umbilical blood flow” [Tiab] OR “arteria umbilicalis” [Tiab] OR "utero-placental"[Tiab]  NOT ("Human Umbilical Vein Endothelial Cells"[Mesh] OR "Umbilicus"[Mesh] OR "Hernia, Umbilical"[Mesh] OR “TUBA”[Tiab] OR “Umbilical hernia”[Tiab] OR “Human Umbilical Vein Endothelial Cells”[Tiab] OR “HUVECs” [tiab] OR “human umbilical arterial endothelial cells”[Tiab] OR “HUAECs”[Tiab] OR “Primary umbilical endometriosis”[Tiab] OR Umbilicus[Tiab] OR “medial umbilical ligament”[Tiab]) | 38,051 | 3,149 |
| 2-Blood Flow | "Blood Flow Velocity"[Mesh] OR "Hemorheology"[Mesh] OR "Blood"[Mesh] OR "blood supply" [Subheading] OR “Blood velocity”[Tiab] OR “Blood flow”[Tiab] OR “arterial flow”[Tiab] OR “impedance to flow”[Tiab] | 1,521,689 | 56,483 |
| 3-Ultra Sonography | "Ultrasonography, Doppler"[Mesh:NoExp] OR “Ultrasonography, Doppler, Pulsed"[Mesh:NoExp] OR "Ultrasonography, Doppler, Duplex"[Mesh:NoExp] OR "Ultrasonography, Doppler, Color"[Mesh:NoExp] OR "Ultrasonography, Prenatal"[Mesh:NoExp] OR "Pulse Wave Analysis"[Mesh] OR "Doppler Effect"[Mesh] OR  “Doppler ultrasound”[Tiab] OR “Doppler Ultrasonography”[Tiab] OR “Doppler sonography”[Tiab] OR “Doppler Duplex”[Tiab] OR “Doppler Color”[Tiab] OR “Doppler Colour”[Tiab] OR “Doppler Pulsed”[Tiab] OR “Pulsed wave Doppler”[Tiab] OR “Continuous wave Doppler”[Tiab] OR “Color Doppler”[Tiab] OR “Doppler mode”[Tiab]  OR "power doppler"[Tiab] OR "conventional Doppler"[Tiab] | 84,294 | 3,149 |
| 4 | 1 + 2 + 3 | 2,109 | 120 |
| 4+ Human filter | NOT (“Animals” [Mesh])  NOT ("Animals"[Mesh] AND "Humans"[Mesh])) | 2,003 | 102 |

**Embase (22 December 2017, updated 10 January 2020)**

| **Concept** | **Search string** | **Results (22.12.17)** | **Results (10.01.20)** |
| --- | --- | --- | --- |
| 1-umbilical | 'umbilical cord'/exp OR 'single umbilical artery'/exp  OR 'umbilical artery'/exp OR 'umbilical vessels':ti,ab OR 'umbilical cord':ti,ab OR 'umbilical cable':ti,ab OR 'umbilical artery':ti,ab OR 'umbilical arteries':ti,ab OR 'umbilical circulation':ti,ab OR 'umbilical blood flow':ti,ab OR 'arteria umbilicalis':ti,ab OR 'utero-placental':ti,ab  NOT ('HUAEC cell line'/exp OR 'umbilicus'/exp OR 'umbilical hernia'/exp OR 'TUBA':ti,ab OR 'Umbilical hernia':ti,ab OR 'Human Umbilical Vein Endothelial Cells':ti,ab OR 'HUVECs':ti,ab OR 'human umbilical arterial endothelial cells':ti,ab OR 'HUAECs':ti,ab OR 'Primary umbilical endometriosis':ti,ab OR Umbilicus:ti,ab OR 'medial umbilical ligament':ti,ab) | 65,685 | 11,423 |
| 2-Blood Flow | 'blood flow velocity'/exp OR 'blood rheology'/exp OR 'blood'/exp OR 'blood flow'/exp OR 'Blood flow':ti,ab OR 'arterial flow':ti,ab OR 'impedance to flow':ti,ab OR 'blood velocity':ti,ab | 2,518,768 | 312,287 |
| 3-Ultra Sonography | 'Doppler ultrasonography'/de OR 'pulsed Doppler ultrasonography'/de OR 'duplex Doppler ultrasonography'/de OR 'color Doppler flowmetry'/de OR 'fetus echography'/de OR 'pulse wave'/de OR 'doppler effect'/de OR 'Doppler ultrasound':ti,ab OR 'Doppler Ultrasonography':ti,ab OR 'Doppler sonography':ti,ab OR 'Doppler Duplex':ti,ab OR 'Doppler Color':ti,ab OR 'Doppler Colour':ti,ab OR 'Doppler Pulsed':ti,ab OR 'Pulsed wave Doppler':ti,ab OR 'Continuous wave Doppler':ti,ab OR 'Color Doppler':ti,ab OR 'Doppler mode':ti,ab OR 'power doppler':ti,ab OR 'conventional Doppler':ti,ab | 124,519 | 23,475 |
| 4 | 1+2+3 | 1734 | 246 |
| 4 + Human filter | NOT ([animals]/lim NOT [humans]/lim) | 1640 | 209 |

**CINAHL (22 December 2017, updated 10 January 2020)**

| **Concept** | **Search string** | **Results (22.12.17)** | **Results (10.01.20)** |
| --- | --- | --- | --- |
| 1-umbilical | MH ("Umbilical Arteries" OR "Umbilical Cord+" OR "Umbilical Veins") OR  TI("umbilical vessels" OR "umbilical cord" OR "umbilical cable" OR "umbilical artery" OR "umbilical arteries" OR "umbilical circulation" OR "umbilical blood flow" OR "arteria umbilicalis" OR "utero-placental") OR  AB("umbilical vessels" OR "umbilical cord" OR "umbilical cable" OR "umbilical artery" OR "umbilical arteries" OR "umbilical circulation" OR "umbilical blood flow" OR "arteria umbilicalis" OR "utero-placental")  NOT (MH ( "Umbilicus" OR "Hernia, Umbilical") OR TI("TUBA" OR "Umbilical hernia" OR "Human Umbilical Vein Endothelial Cells" OR "HUVECs" OR "human umbilical arterial endothelial cells" OR "HUAECs" OR "Primary umbilical endometriosis" OR Umbilicus OR "medial umbilical ligament") OR AB("TUBA" OR "Umbilical hernia" OR "Human Umbilical Vein Endothelial Cells" OR "HUVECs" OR "human umbilical arterial endothelial cells" OR "HUAECs" OR "Primary umbilical endometriosis" OR Umbilicus OR "medial umbilical ligament") | 5,105 | 1,466 |
| 2-Ultra Sonography | MH ("Ultrasonography, Doppler" OR "Ultrasonography, Doppler, Pulsed" OR "Ultrasonography, Doppler, Duplex" OR "Ultrasonography, Doppler, Color" OR "Ultrasonography, Prenatal") OR  TI("Doppler ultrasound" OR "Doppler Ultrasonography" OR "Doppler sonography" OR "Doppler Duplex" OR "Doppler Color" OR "Doppler Colour" OR "Doppler Pulsed" OR "Pulsed wave Doppler" OR "Continuous wave Doppler" OR "Color Doppler" OR "Doppler mode" OR "power doppler" OR "conventional Doppler") OR  AB("Doppler ultrasound" OR "Doppler Ultrasonography" OR "Doppler sonography" OR "Doppler Duplex" OR "Doppler Color" OR "Doppler Colour" OR "Doppler Pulsed" OR "Pulsed wave Doppler" OR "Continuous wave Doppler" OR "Color Doppler" OR "Doppler mode" OR "power doppler" OR "conventional Doppler") | 16,923 | 2,660 |
| 3 | 1+2 | 833 | 101 |
| 4 | Database filter - Exclude Medline | 89 | 25 |
| 5 | Human filter | 89 | 19 |

**Cochrane CENTRAL (22 December 2017, updated 14 January 2020)**

| **Concept** | **Search string** | **Results (22.12.17)** | **Results (14.01.20)** |
| --- | --- | --- | --- |
| 1-umbilical | [mh "Umbilical Cord"] OR "umbilical vessels":ti,ab OR "umbilical cord" :ti,ab OR "umbilical cable" :ti,ab OR "umbilical artery" :ti,ab OR "umbilical arteries" :ti,ab OR "umbilical circulation" :ti,ab OR "umbilical blood flow" :ti,ab OR "arteria umbilicalis" :ti,ab OR "utero-placental":ti,ab | 1,759 | 3,405 |
| 2-Ultra Sonography | [mh "Ultrasonography, Doppler"] OR [mh "Ultrasonography, Doppler, Pulsed"] OR [mh "Ultrasonography, Doppler, Duplex"] OR [mh "Ultrasonography, Doppler, Color"] OR [mh "Ultrasonography, Prenatal"] OR [mh "Pulse Wave Analysis"] OR [mh "Doppler Effect"] OR  "Doppler ultrasound":ti,ab OR "Doppler Ultrasonography":ti,ab OR "Doppler sonography":ti,ab OR "Doppler Duplex":ti,ab OR "Doppler Color":ti,ab OR "Doppler Colour":ti,ab OR "Doppler Pulsed":ti,ab OR "Pulsed wave Doppler":ti,ab OR "Continuous wave Doppler":ti,ab OR "Color Doppler":ti,ab OR "Doppler mode":ti,ab  OR "power doppler":ti,ab OR "conventional Doppler":ti,ab | 5,165 | 6,480 |
| 3 | 1 + 2 | 143 | 175 |
| 4 | Trials only | 129 | 168 |
|  | *Limit to yr=”2017 – 2020”* | N/A | 17 |

**Global Index Medicus (21 December 2017, updated 10 January 2020)**

| **Concept** | **Search string** | **Results (21.12.17)** | **Results (10.01.20)** |
| --- | --- | --- | --- |
| 1-umbilical | mh:("Umbilical Arteries" OR "Umbilical Cord" OR "Umbilical Veins" OR "Single Umbilical Artery") OR  ti: ("umbilical vessels" OR "umbilical cord" OR "umbilical cable" OR "umbilical artery" OR "umbilical arteries" OR "umbilical circulation" OR "umbilical blood flow" OR "arteria umbilicalis" OR "utero-placental") OR  ab: ("umbilical vessels" OR "umbilical cord" OR "umbilical cable" OR "umbilical artery" OR "umbilical arteries" OR "umbilical circulation" OR "umbilical blood flow" OR "arteria umbilicalis" OR "utero-placental") | 3,563 | 376 |
| 2-Ultra Sonography | mh:("Ultrasonography, Doppler" OR "Ultrasonography, Doppler, Pulsed" OR "Ultrasonography, Doppler, Duplex" OR "Ultrasonography, Doppler, Color" OR "Ultrasonography, Prenatal" OR "Pulse Wave Analysis" OR "Doppler Effect") OR  Ti:("Doppler ultrasound" OR "Doppler Ultrasonography" OR "Doppler sonography" OR "Doppler Duplex" OR "Doppler Color" OR "Doppler Colour" OR "Doppler Pulsed" OR "Pulsed wave Doppler" OR "Continuous wave Doppler" OR "Color Doppler" OR "Doppler mode" OR "power doppler" OR "conventional Doppler") OR  Ab: ("Doppler ultrasound" OR "Doppler Ultrasonography" OR "Doppler sonography" OR "Doppler Duplex" OR "Doppler Color" OR "Doppler Colour" OR "Doppler Pulsed" OR "Pulsed wave Doppler" OR "Continuous wave Doppler" OR "Color Doppler" OR "Doppler mode" OR "power doppler" OR "conventional Doppler") | 89,228 | 677 |
| 3 | **1+2** | 3724 | 249 |
| 3 + Database filter | AND ( db:("LILACS" OR "WHOLIS" OR "WPRIM" OR "IMSEAR" OR "IMEMR" OR "AIM")) | 200 | 249 |
