# Supplementary material for: Prevalence of abnormal umbilical arterial flow on Doppler ultrasound in low-risk and unselected pregnant women: a systematic review
Source: Reprod Health. 2021 Feb 12;18:38. doi: 10.1186/s12978-021-01088-w (PMC7881445; doi:10.1186/s12978-021-01088-w)
Supplement: Supplementary file 4 — Additional file 4: Appendix S4. Quality assessments. [file 12978_2021_1088_MOESM4_ESM.docx]

**Appendix S4. Quality assessment for different study types**

**Cross-sectional studies^[[1]](#footnote-1)^**

| **Study** | **Score (possible range 0 – 10)** | **Overall quality assessment** |
| --- | --- | --- |
| Adali 2009 | 4 | Unsatisfactory |
| Adali, 2012 | 7 | Good |
| Aksoy, 2015 | 7 | Good |
| Ayoola 2016 | 5 | Satisfactory |
| Bolz, 2013 | 4 | Unsatisfactory |
| Bustos 2014 | 5 | Satisfactory |
| Ferdousi, 2013 | 4 | Unsatisfactory |
| Kiserud, 2006 | 8 | Good |
| Santos, 2016 | 7 | Good |
| Skotnicki, 2000 | 6 | Satisfactory |
| Souka, 2012 | 9 | Very good |
| Welsh, 2012 | 7 | Good |

| **Study** | **Score (possible range 0 – 10)** | **Overall quality assessment** |
| --- | --- | --- |
| Alptekin 2017 | 6 | Satisfactory |
| Guo 2019 | 3 | Unsatisfactory |
| Lobmaier, 2012 | 5 | Satisfactory |
| Raio, 2003 | 8 | Good |
| Tan, 2005 | 6 | Satisfactory |
| Thuring, 2012 | 6 | Satisfactory |
| Yildiz, 2011 | 5 | Satisfactory |

**Case-control studies^[[2]](#footnote-2)^**

**Cohort studies^[[3]](#footnote-3)^**

| **Study** | **Score (possible range 0 – 13)** | **Overall quality asessment** |
| --- | --- | --- |
| Acharaya 2005 | 8 | Satisfactory |
| Arstrom 1989 | 8 | Satisfactory |
| Beattie, 1989 | 9 | Good |
| Benzer, 2015 | 7 | Satisfactory |
| Da Costa, 2005 | 7 | Satisfactory |
| Drukker 2020 | 9 | Good |
| Fay-1994 | 6 | Satisfactory |
| Figueira, 2016 | 8 | Satisfactory |
| Flo, 2010 | 7 | Satisfactory |
| Helbig, 2013 | 8 | Satisfactory |
| Hutter 1991 | 8 | Satisfactory |
| Jorge Neto, 2016 | 7 | Satisfactory |
| Jorge Neto, 2016b | 6 | Satisfactory |
| Kehila, 2017 | 6 | Satisfactory |
| Nkosi, 2019 | 6 | Satisfactory |
| Ome‑Kaius, 2017 | 9 | Good |
| Owen 2003 | 7 | Satisfactory |
| Widnes 2018 | 8 | Satisfactory |
| Yoon 1993 | 6 | Satisfactory |

**Randomised controlled trials^[[4]](#footnote-4)^**

| **Study** | **Random sequence allocation** | **Allocation concealment** | **Blinding participants and personnel** | **Blinding of outcome assessment** | **Incomplete outcome reporting** | **Selective outcome reporting** | **Other bias** |
| --- | --- | --- | --- | --- | --- | --- | --- |
| Atkinson 1994 | low | low | low | low | low | low | unclear |
| Davies, 1992 | unclear | low | high | high | low | unclear | low |
| Doppler French Study, 1997 | unclear | low | high | high | low | unclear | low |
| Mason, 1993 | low | low | high | high | low | unclear | unclear |

1. Newcastle-Ottawa scale, adapted for cross-sectional studies. Unsatisfactory (0 to 4), Satisfactory (5-6), Good (7-8), Very good (9-10) [↑](#footnote-ref-1)
2. Newcastle-Otttawa scale, adapted for case-control studies. Unsatisfactory (0 to 4), Satisfactory (5-6), Good (7-8), Very good (9-10) [↑](#footnote-ref-2)
3. Newcastle-Ottawa scale, adapted for cohort studies. Unsatisfactory (0 to 5), Satisfactory (6-8), Good (9-11), Very good (12-13) [↑](#footnote-ref-3)
4. Cochrane risk of bias tool [↑](#footnote-ref-4)
